# Evaluation of Porous (Poly(lactide-*co*-glycolide)-*co*-(ε-caprolactone)) Polyurethane for Use in Orthopedic Scaffolds

**DOI:** 10.3390/molecules29040766

**Published:** 2024-02-07

**Authors:** Gaëlle Savin, Océane Sastourne-Array, Sylvain Caillol, Audrey Bethry, Michel Assor, Ghislain David, Benjamin Nottelet

**Affiliations:** 1 ICGM, Univ Montpellier, CNRS, ENSCM, 34000 Montpellier, France; gaelle.savin@enscm.fr (G.S.); ghislain.david@enscm.fr (G.D.); 2 IBMM, Univ Montpellier, CNRS, ENSCM, 34000 Montpellier, France; oceane.sastourne-arrey@etu.umontpellier.fr (O.S.-A.); audrey.bethry@umontpellier.fr (A.B.); 3 Arthrocart Biotech, 13000 Marseille, France; michel.assor@arthrocart.com; 4 Department of Pharmacy, Nîmes University Hospital, University Montpellier, 30900 Nimes, France

**Keywords:** porous scaffold, poly(ester-urethane), poly(lactide-*co*-glycolide), poly(e-caprolactone), particulate leaching

## Abstract

To develop an orthopedic scaffold that could overcome the limitations of implants used in clinics, we designed poly(ester-urethane) foams and compared their properties with those of a commercial gold standard. A degradable poly(ester-urethane) was synthetized by polyaddition between a diisocyanate poly(ε-caprolactone) prepolymer (PCL di-NCO, *M_n_* = 2400 g·mol^−1^) and poly(lactic-*co*-glycolic acid) diol (PLGA, *M_n_* = 2200 g·mol^−1^) acting as a chain extender. The resulting high-molecular-weight poly(ester-urethane) (PEU, *M_n_* = 87,000 g·mol^−1^) was obtained and thoroughly characterized by NMR, FTIR and SEC-MALS. The porous scaffolds were then processed using the solvent casting (SC)/particle leaching (PL) method with different NaCl crystal concentrations. The morphology, pore size and porosity of the foams were evaluated using SEM, showing interconnected pores with a uniform size of around 150 µm. The mechanical properties of the scaffolds are close to those of the human meniscus (Ey = 0.5~1 MPa). Their degradation under accelerated conditions confirms that incorporating PLGA into the scaffolds greatly accelerates their degradation rate compared to the gold-standard implant. Finally, a cytotoxicity study confirmed the absence of the cytotoxicity of the PEU, with a 90% viability of the L929 cells. These results suggest that degradable porous PLGA/PCL poly(ester-urethane) has potential in the development of meniscal implants.

## 1. Introduction

The knee menisci, discs of fibrocartilage interposed between the tibia and the condyles of the femur, are essential in load bearing and load distribution, shock absorption, stabilization and lubrication of the knee joint [1]. However, meniscal tears resulting from aging and joint injury often occur with little chance of spontaneous repair due to the limited capacity of articular cartilage to restore. These defects cause join pain and the loss of mobility [2]. Current treatments include non-pharmacologic (e.g., structured exercise) and pharmacologic (e.g., nonsteroidal anti-inflammatory drugs) treatments [3], as well as direct intra-articular hyaluronic acid injections [4]. Patients with persistent pain and functional loss are candidates for total or partial knee replacement. Meniscal tears may be treated with the resection, repair or replacement of the damaged tissue [5]. In particular, when fragments interfere with joint movement, arthroscopic partial meniscectomy (APM) procedures are performed to remove fragments of the meniscus that stick out into the joint. This surgery results in instant pain relief and the restoration of knee function [6]. APM leads to accelerated cartilage degradation in the long term due to increased peak stresses acting on the articular cartilage [2]. Months, or years, after, patients suffer from the development of degenerative changes and osteoarthritis (OA) with articular cartilage degradation [3]. This “post-meniscectomy syndrome” leads to pain and the worsening of symptoms due to the increased contact stress within the joint [7]. Despite this drawback, APM remains the most performed meniscal treatment as a result of a more active and older population [8,9]. Increasing awareness on meniscus loss has led to the search for implants allowing cellular growth [8], but to date, only two implants were translated into clinical use: the Collagen Meniscus Implant (Menaflex, formerly called CMI, ReGen Biologics, Franklin Lakes, NJ, USA), and Actifit^®^ (Orteq Ltd., London, UK). These two scaffolds are biodegradable and promote colonization by endogenous cells that differentiate and regenerate fibrocartilage. A recent meta-analysis highlighted that both approaches yield favorable clinical outcomes, resulting in symptom improvement and improved meniscus function. Furthermore, they demonstrate a relatively low failure rate, with the CMI^®^ showing a 7% failure rate and Actifit^®^ showing a 9% failure rate. Moreover, the surgically prepared site for these implants must extend at least into the red/white zone of the meniscus to provide sufficient vascularization [10].

The porous biological scaffold CMI is intended for use in surgical procedures for the reinforcement and repair of soft tissue injuries. The scaffold is composed of 3% of glycosaminoglycan (GAG) [11] and 97% of a type I collagen from bovine Achilles tendons [12]. This implant exhibits unwanted folding during surgery due to its poor mechanical properties, leading to an uneven pressure, poorer mechanical properties than native meniscus, and a too-fast degradation in vivo to ensure sufficient time for cellular growth and new meniscus tissue formation [10,13,14,15]. Actifit^®^ is a polyurethane-based scaffold indicated for the repair of painful, irreparable partial meniscal defects. The highly porous scaffold acts as a template for the proliferation and organization of cells with extracellular matrix [16]. It is synthetized from poly(ε-caprolactone) (PCL), butanediol (BDO) and butanediisocyanate (BDI), allowing for strength and flexibility. This scaffold suffers from slow degradation (5 years) [17,18]. Tissue regeneration under the support of scaffolds is a rather uncertain process, affected by cells and their interaction with the scaffolds. It is therefore difficult to predict the optimal degradation kinetics for a meniscal scaffold. However, it is recognized that scaffolds exhibiting too-slow degradation tend to inhibit efficient tissue regeneration and continuously stimulate the host’s foreign body response [19]. Of note, both implants are porous materials to cope with the widely recognized requirements of scaffolds; they must have an optimal pore size and shape, and optimal pore size distribution and interconnectivity to ensure correct cell attachment, proliferation and tissue ingrowths [20]. According to literature reports, pore sizes ranging from 100 µm to 300 µm and a porosity of 80% are necessary [21,22,23].

To address some of the limitations of the aforementioned scaffolds, we propose in this work the use of poly(ester-urethane)s (PEUs) of soundly selected compositions to design porous scaffolds, reach suitable mechanical properties of meniscal repair, and ensure faster degradation compared to the PCL-based slow-degrading Actifit^®^. PEUs are among the most used biomaterials in the biomedical field [24]. They are considered bioabsorbable networks, showing excellent mechanical and physical properties, as well as good blood compatibility [25]. Thermoplastic PEU elastomers offer tunable mechanical properties and the fine control of the degradation rate [26]. In particular, research has focused on biodegradable and biocompatible PEUs based on copolyesters of ε-caprolactone, D,L-lactide, glycolide associated with various low-molecular-weight diisocyanate [27,28,29]. PCL, poly(D,L-lactide) (PLA) and poly(lactic-*co*-glycolic acid) (PLGA) act as soft segments in the polymer chain, whereas diisocyanates, typically hexamethylene diisocyanate (HDI) and pentamethylene diisocyanate (PDI), are considered hard segments due to urethane linkage, yielding to hydrogen bonding and physical interactions. The mechanical properties of PEU can therefore be tailored depending on the hard and soft segment molar ratio, the nature of these segments and the process used to yield scaffolds. Among these processes, solvent casting (SC)/particle leaching (PL) is largely used with polyurethanes and consists of loading particles with specific diameters in a polymer solution. After solvent evaporation, the particles entrapped in the polymer matrix are finally leached out in water to leave a porous structure [20,21,22].

**Figure 1:**
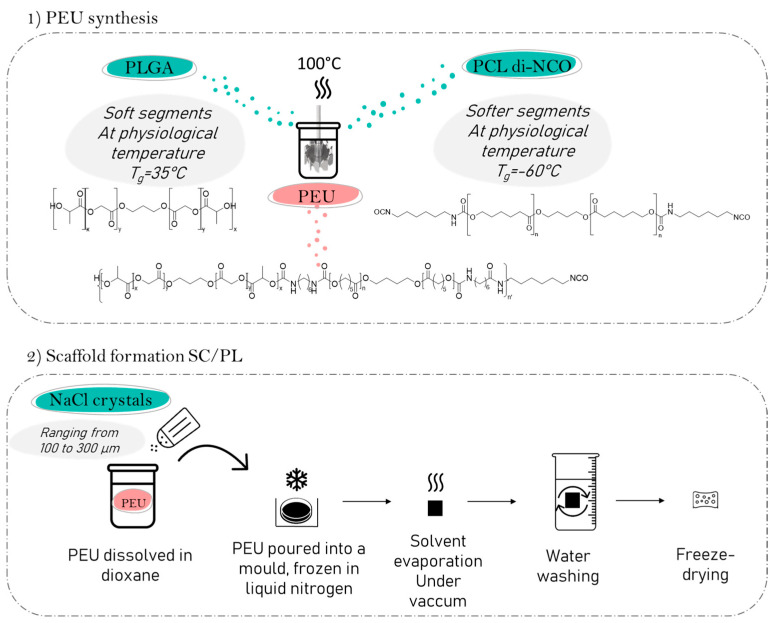
Synthesis of poly(ester-urethane) from PCL and PLGA and formation of PEU scaffold via solvent casting/salt leaching.

In this work, our aim is to take advantage of PEU’s unique properties and SC/PL to design new biodegradable porous scaffolds suitable for knee meniscus repair. To this aim, we first prepare a PEU based on PLGA and PCL blocks acting as soft/high glass temperature (*T_g_*) and softer/low *T_g_* segments, respectively. The selected ratio of LA/GA in the PLGA blocks, the molecular weight of each block and the equimolar composition of PLGA-diol and PCL-diisocyanate chosen to design our PEU allow for faster degradation while maintaining suitable mechanical properties. We demonstrate that high-molecular-weight PEU is obtained by polyaddition and can be used to yield porous scaffolds through the SC/PL technique (Figure 1). The mechanical properties, the cytotoxicity, and the degradation of the scaffolds are studied and compared to those of a commercial Actifit^®^ implant.

## 2. Results and Discussion

### 2.1. PLGA Synthesis

**Figure 2:**
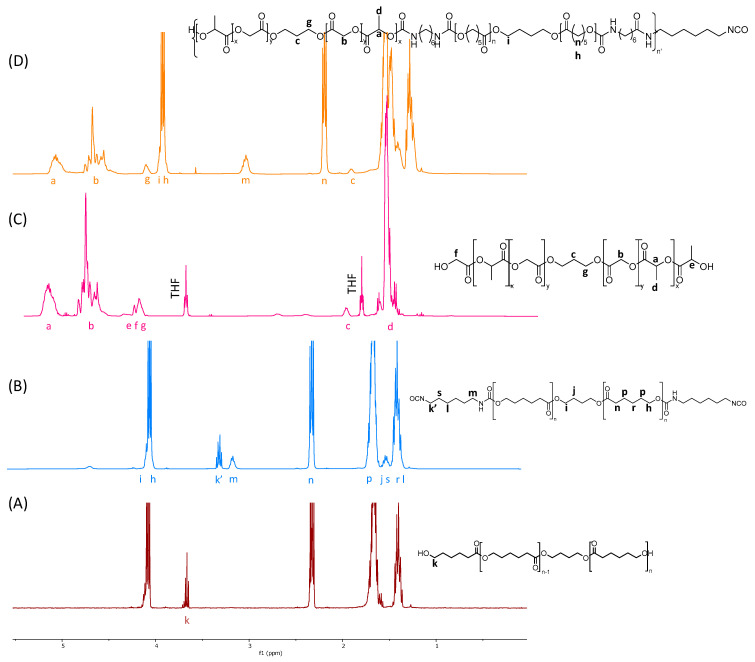
^1^H NMR (400 MHz, CDCl_3_) spectra of (**A**) PCL diol, (**B**) prepolymer PCL di−NCO, (**C**) PGLA, (**D**) PCL/PLGA PEU.

**Figure 3:**
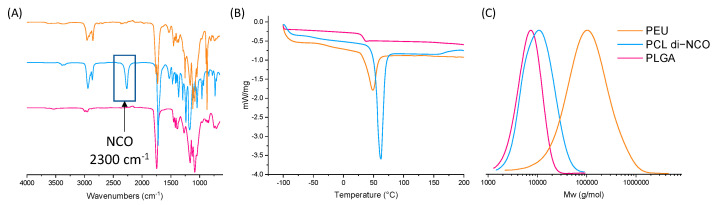
Characterizations of the polyesters and PEU (**A**) FTIR spectra, (**B**) DSC thermograms, (**C**) SEC chromatograms (THF).

The PLGA diol was synthetized by the ROP of an equimolar ratio of D,L-lactide and glycolide initiated by PDO and with a targeted molecular weight of 2000 g·mol^−1^. The polymerization yield was close to 90%. The ^1^H NMR spectrum allowed us to determine the average molar mass and the ratio of the monomer units. The peaks **a** and **d** at 5.2 ppm and 1.4 ppm were attributed to the methine and methyl groups of the lactic units, and the peak **b** at 4.8 ppm was attributed to the methylene of the glycolic units (Figure 2C). These peaks were used to calculate the ratio of the lactic units to glycolic units that was equal to 50:50 in agreement with the feed ratio. An average molar mass of 2200 g·mol^−1^ was also calculated by ^1^H NMR using the peaks at 5.2 ppm and 4.8 ppm. The molecular weight was also measured by SEC analysis with a value of 3900 g·mol^−1^. The MALDI TOF analyses (Figure S3) confirmed the structure of the PLGA, with a statistical distribution of the lactic unit (*M* = 72 g·mol^−1^) and glycolic unit (*M* = 58 g·mol^−1^). The thermogram obtained by the TGA analysis of PLGA (Figure S4) showed that the decomposition of the polymer chains occurred at 173 °C (5% of mass loss), in accordance with the values found in the literature [30,31]. A typical DSC curve of the PLGA is shown in Figure 3 with a *T_g_* at 35 °C, in agreement with the values in the literature [16].

### 2.2. PCL di-NCO Prepolymer Synthesis

The PCL-diol with a molar mass of 2400 g·mol^−1^ according to the ^1^H NMR calculation was end-capped with an excess of HDI at 80 °C to form the prepolymer. The derivatization of the PCL-diol towards PCL di-NCO was monitored by ^1^H NMR spectroscopy. The spectra showed the complete disappearance of the signal from the CH_2_ close to the hydroxyl groups of the PCL-diol at 3.7 ppm and the appearance of two new signals at 3.20 and 3.40 ppm corresponding to the methylene in the α-position of the urethane functional group, and the methylene at the α-position of the isocyanate functional group (Figure 2B).

The synthesis of the prepolymer was further confirmed by FTIR spectroscopy, with the spectra showing a sharp peak at 2300 cm^−1^ corresponding to the stretching vibration of the isocyanate group N=C=O. The complete removal of the non-reacted HDI was ensured by the precipitation of the prepolymer in cold pentane to avoid side reactions between the PLGA diol and residual HDI. The prepolymer was finally dried under vacuum for 48 h at 40 °C to ensure the complete removal of the residual traces of the free isocyanates. The efficiency of the purification process was confirmed by ^1^H NMR, where the intensity of the peak corresponding to the NCO-C**H_2_**-CH at 3.30 ppm (**k’**) was equal to the one of CH_2_-C**H_2_**-NHCOO at 3.10 ppm (**m**). Moreover, the thermogram of the PCL di-NCO (Figure S4) showed that the decomposition of the polymer chains occurred at 300 °C (5% mass loss), underlining the good stability of the polymer at physiological temperature.

### 2.3. Poly(ester-urethane) Synthesis

The isocyanate-terminated prepolymer was extended with the PLGA-diol at a 1.1:1 ratio in a dry glass reactor purged with nitrogen. The reaction was conducted with a mechanical stirring blade at 100 °C for 1 h with a minimum of distilled dioxane to ensure a viscosity compatible with the process. A typical PEU ^1^H NMR spectrum is displayed in Figure 2D.

The disappearance of the isocyanate peak at 3.3 ppm shows that the PEU is terminated by hydroxyl moieties, with no isocyanate residues still present in the PEU. By the integration of H_b_ and H_n_, the ^1^H NMR spectrum shows a 50:50 ratio of PCL:PLGA, in agreement with the feed ratio.

The comparative FTIR spectra of the PEU, PLGA-diol and the PCL di-NCO prepolymer are displayed in Figure 3A. The vibrations of the -C=O group of the PCL backbone, PLGA and urethane bonds can be observed between 1730 and 1720 cm^−1^. The band at 3500 cm^−1^ indicates the presence of hydroxyl groups in the copolymer PLGA. The presence of the -CH_2_ units in the polymer backbone is revealed by the set of peaks occurring between 2940 and 2920 cm^−1^ and 2860 and 2850 cm^−1^. For the PEU, a broad stretching band between 3500 and 3700 cm^−1^ is present, corresponding to the N-H bond of the urethane groups formed during the reaction. Moreover, the disappearance of the stretching band at 2300 cm^−1^, characteristic of isocyanate groups, indicates the full conversion of the isocyanate groups after one hour.

The DSC thermograms, as seen in Figure 3B, of the PEU indicate a melting temperature (*T_m_*) at approximatively 50 °C and a *T_g_* at −50 °C. According to the thermograms of PLGA (*T_g_* at 35 °C and no *T_m_*), no *T_g_* of the PLGA blocks is visible on the PEU thermograms as this transition is in the melting peak of the polymer that corresponds to the melting of the PCL blocks. The observed *T_g_* at −50 °C corresponds to the *T_g_* of the PCL blocks. The H bonds from the urethane groups of the PEU are responsible for the increased interactions of the polymer chains, leading to an increase in the *T_g_*. The thermal decomposition of the PEU (5% mass loss), as seen in Figure S4, occurred at 227 °C, which underlines the stability of the PLGA copolymer (T_d_ 5% = 173 °C) and PCL (T_d_ 5% = 300 °C) mixture.

To compare our PEU with the one used in Actifit^®^, an SEC analysis was performed on a sample of this commercial scaffold composed of PCL polyurethane. Molecular weights of *M_n_* = 104,000 g⋅mol^−1^ and *M_w_* = 316,000 g⋅mol^−1^ (Ð = 3) were obtained by SEC-MALS considering a dn/dc = 0.083 for PCL (Figure S5). Of note, two populations were observed with a clear shoulder on the chromatogram. This was not the case for our PEU, with an average number molar mass of *M_n_* = 87,000 g⋅mol^−1^, as determined by the SEC-MALS, with a narrower dispersity of 1.6 (see dn/dc values in Table S1). This high molecular weight is essential to target the compression modulus close to the human meniscus modulus.

### 2.4. Scaffold Preparation

**Figure 4:**
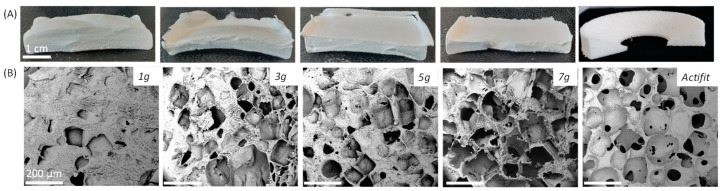
Characterization of the scaffolds with different amount of salt. (**A**) Macroscopic micrographs, (**B**) SEM micrographs as a function of salt content, showing the pore shape and density of pores. Scale bar of the microscopy images corresponds to 200 µm.

An optimal scaffold aiming to repair menisci should contain large pores for cell ingrowth, and should have good mechanical properties and a high porosity. It has been demonstrated that the optimal ingrowth of fibrocartilaginous tissue takes place for pore sizes in the range 100–300 µm [24]. Therefore, NaCl was sieved at 100–300 µm to ensure pore sizes were in this range, a common step described in other studies [32,33,34,35,36]. The PEU was solubilized in dioxane, as we selected the same solvent used for the PEU synthesis. DMSO is commonly used for the solvent casting method [1,34,37], but in our study, we utilized a solvent with a relatively low boiling point (<150 °C) to easily remove it under vacuum. The optimal PEU concentration in dioxane was determined to obtain the viscosity necessary to homogeneously disperse the salt crystals and avoid sedimentation. An optimal concentration is also important during the evaporation step to prevent the creation of cavities in the scaffold due to a high solvent concentration. Following preliminary tests, the dioxane concentration was fixed at 30 wt% (viscosity η = 458 ± 22 Pa·s^−1^ at 25 °C, see Figure S6). In the literature, this concentration ranges from 5 to 60%, depending on many factors, such as the molar mass of the polymer, the nature of the solvent and the addition of a solvent in which the polymer is insoluble [17,32,34,36,37,38,39,40]. We first studied the influence of the NaCl concentration and tested four concentrations at 1, 3, 5 and 7 g of NaCl per gram of PEU. After the freezing and evaporation of dioxane, the porous scaffolds were characterized in terms of the pore size, porosity and mechanical properties (Figure 4). The results are summarized in Table 1.

All the materials were obtained in the form of white foams (Figure 4A). The analysis of the SEM images led to the calculation of an average pore size of around 150 µm for the foams obtained with 3, 5 and 7 g of salt per gram of PEU (Figure 4B). Two populations of pores are observed: one major population with diameter around 150 µm that agrees with the size of the NaCl crystals introduced (100–300 µm) in the PEU solution, and one population at 50 µm that corresponds to the interconnections between the pores of 100 µm diameter (see Figure S8). Of note, for all three of these foams, the porosity was between 36% and 72%, which is close to the value measured for Actifit^®^ that has slightly larger pores of about 240 µm. For the foam with only 1 g of salt per gram of PEU, a large discrepancy of the pore sizes was observed, as shown by the large standard deviation. For this last foam, the average pore size of 111 µm was measured, with a major population at 50 µm due to the interconnectivity, and a second one between 100 and 300 µm. The low salt concentration does not allow for the formation of enough large pores in this case, leading to a lower porosity of 36%. This low porosity and these small pores do not meet the requirements for cell proliferation [29], which disqualify this last sample as a potential scaffold.

**Figure 5:**
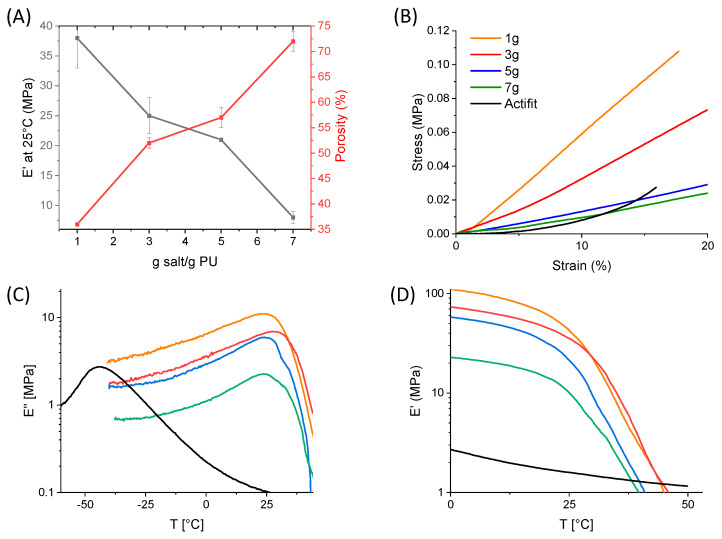
(**A**) Influence of salt concentration on storage modulus at 25 °C and on the overall porosity. (**B**) Stress/strain compression curve at 20 % at room temperature for the different scaffolds, (**C**,**D**) storage modulus E′ and loss modulus E″ of scaffolds as a function of salt content and temperature (dynamic mechanical analysis performed at f = 1 Hz).

Following these morphological characterizations, the mechanical properties of the scaffolds were studied. As expected, the storage modulus decreases as the salt concentration increases (Figure 5A). Indeed, salt crystals are more interconnected when their concentration increases, leading to weaker mechanical properties. This phenomenon was comparable with the results reported by Gubanksa et al. [20] and Bil et al. [41]. The Young’s modulus values are in accordance with the ones found in the literature, ranging from 200 kPa to 5 MPa [17,32,33,35,39,40]. This large range has a direct impact on the flexibility of the foams. Dynamic mechanical analyses were also performed at 1 Hz, confirming these results. Storage moduli between 4.5 and 1.5 MPa were obtained at 37 °C for the PEU foams by increasing the salt concentration, compared to 1.3 MPa for Actifit^®^. The storage modulus of the PEU scaffolds agreed with the modulus of the human meniscus (E_y_ = 0.5–1 MPa).

Of notice, in addition to the value of the compression modulus, the mechanical behaviors of the four foams synthetized and Actifit^®^ are also different. On the typical stress/strain curves (Figure 5B), we can observe a drastic increase in stress after 8% strain for the Actifit^®^ scaffold, whereas the foams with 1 g of salt/g PEU exhibit a constant stress/strain ratio. For the foams with 3 g, 5 g and 7 g of salt/g PEU, there is a slight increase in the stress after a 4% strain. This could be explained by the pore size repartition. Actifit^®^ exhibits two sizes of pores, interconnected between each other. The narrow ones are the first to be compressed, leading to a low stress/strain ratio. The bigger pores are then compressed, leading to the strain increasing at a faster rate than stress. On the contrary, for the foams with 3, 5 or 7 g of salt/g PEU, the interconnectivity is less important, showing no drastic mechanical change in the stress/strain ratio.

DMA was then performed to investigate the viscoelastic properties of the synthesized PEU through the storage modulus (E′) and loss modulus (E″) measurements between −60 °C and 60 °C (Figure 5C,D). The four scaffolds exhibit similar T_α_ (24 °C), which is expected since the PEU remains the same in the four scaffolds. This T_α_, found at the peak of the loss modulus, is relatively close to the *T_g_* = 35 °C of PLGA, and those two values can be linked since the frequency used is 1 Hz. Regarding Actifit^®^, a T_α_ at −43 °C is observed, in accordance with the *T_g_* at −50 °C of the PCL-based PEU used in this medical device. Despite the difference in the *T_g_* of the PEU scaffolds and Actifit^®^, the materials exhibit similar mechanical properties at 37 °C. As for the Young’s modulus, the storage modulus increases as the salt concentration decreases with values from 6.6 to 1.6 MPa at 37 °C (Table 1), this last value being close to that of Actifit^®^.

### 2.5. Cytotoxicity

**Figure 6:**
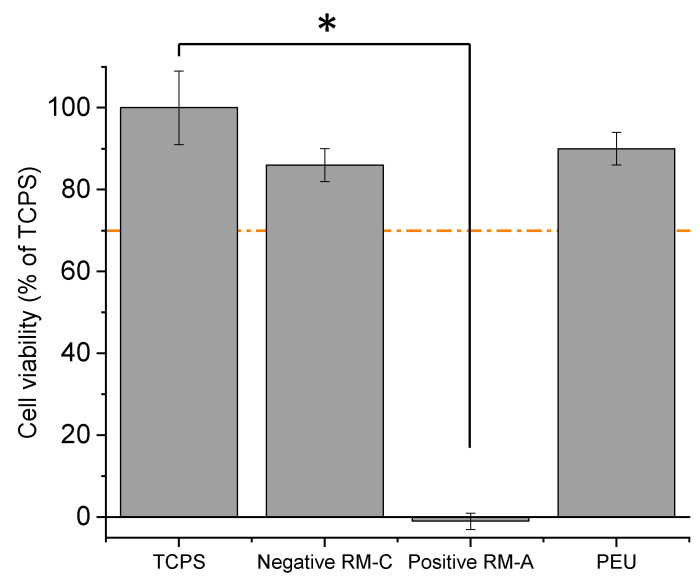
Biological compatibility study of samples: PEU film was not cytotoxic after 72 h of contact with L929 cells compared to the negative RM-C, positive RM-A (PU with ZDEC) and TCPS. Orange dotted line corresponds to 70% of TCPS. Data are expressed as means ± SD and correspond to measurements with *n* = 3. Statistical analyses use a Kruskal–Wallis test followed by a Dunn post hoc test (threshold of significance * *p* < 0.05).

To evaluate the cytocompatibility of our PEU, a cytotoxicity study was conducted. The CellTiter-Glo^®^ method uses the firefly luciferase reaction to determine the relative numbers of living cells. Indeed, metabolically active cells produce ATP, which is required by the luciferase reaction. By luminescence, we measure the amount of light produced, which is proportional to the number of viable cells. The PEU was compared to tissue culture polystyrene (TCPS) and two controls, positive RM-A and negative RM-C, in agreement with the European standard ISO 10993 (https://www.iso.org/standard/36406.html, accessed on 15 January 2024). As seen in Figure 6, the percentage of the viability of the L929 cells in contact with the PEU films was 90% and 86% for the HDPE films (negative RM-C), which is higher than the percentage fixed by the European standard ISO 10993 (70% with respect to TCPS control, see orange dot lines). This result confirms that the PEU film obtained is not cytotoxic and can be used for cell-contacting biomedical applications.

### 2.6. Degradation

**Figure 7:**
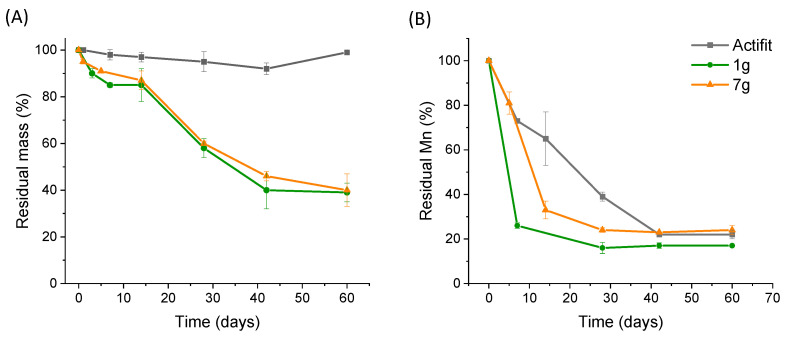
Accelerated in vitro degradation (HCl 0.1 M, 37 °C, pH = 1) of Actifit^®^ scaffold and foams synthetized with 1 g and 7 g of salt/g PEU with (**A**) evolution of residual mass over time and (**B**) evolution of residual molecular weight over time. Data are expressed as means ± SD and correspond to measurements with *n* = 3.

One month of accelerated degradation was conducted in HCl at pH = 1 at 37 °C. Figure 7A shows that after 42 days, the Actifit^®^ scaffold lost only 8% of its mass. It was compared with the two scaffolds obtained with the lowest (1 g) and highest (7 g) quantities of salt. In both cases, and independent of the pore sizes and average porosity, the PLGA/PCL-based PEU scaffolds lost 60% of their mass after 42 days. This validates our initial design targeting faster-degrading porous PEU scaffolds, with the PLGA blocks contained in the PEU enabling a faster degradation rate due to a lower hydrophobicity compared to PCL and thus the faster hydrolysis of the ester bonds. The hydrolysis of semi crystalline polyesters first takes place in the amorphous regions, followed by the crystalline phase degradation, as confirmed by other works reporting the degradation of poly(ester-urethane) [26,29,42].

Regarding the evolution of the average molar mass over time, the PEU scaffolds lost 80% of their molar mass after 10 days, whereas Actifit^®^ needed 40 days to reach the same level (see Figure 7B). This difference is likely due to the PLGA blocks of our PEU that degrade faster into small water-soluble oligomers that can diffuse out of the matrix, which explains the rapid mass loss. On the contrary, Actifit^®^ contains only PCL segments that degrade more slowly and lead to oligomers that for a similar degree of polymerization are less soluble than the PLGA ones and therefore are less prone to diffuse out of the scaffold. Overall, this accelerated degradation compared to the commercial gold standard is in favor of an improved colonization of cells and integration into tissues [43]. Our PEU scaffold exhibited a fourfold increase in the degradation rate when compared to Actifit^®^, as evidenced by the reduction in the molecular mass from 40 days to 10 days under accelerated conditions. Although promising, these results will have to be confirmed by in vivo studies to confirm that our PEU scaffolds present a suitable degradation profile, not too fast to avoid the too-quick loss of mechanical properties, but not too slow to avoid chronic foreign body response.

## 3. Materials and Methods

### 3.1. Materials

D,L-lactide (Lot no.: 2009002968) and glycolide (Lot no.: 1210002433) were purchased from Corbion (Gorinchem, The Netherlands). Tin (II) 2-ethylhexanoate (92.5–100%, Lot no.: 1003287404), poly(ε-caprolactone) diol (PCL, *M_n_* = 2000 g.mol^−1^, WXBD4206V), 1,3-propanediol (>98%, Lot no.: S768868143) were provided by Sigma Aldrich (St-Quentin Fallavier, France) and used as received. 1,4-Dioxane (ACS-Reagent, Lot no.: M1600), distilled in the presence of CaH_2_, was provided by Honeywell (Offenbach, Germany). Diethyl ether (Batch Number: V1G060031H), n-pentane (Batch Number: V9C059249D), dichloromethane (Batch Number: V1M602021N) and sodium chloride (Batch Number: V1L077212C) were provided by Carlo Erba Reagents (Val de Reuil, France). Hexamethylene diisocyanate (HDI; >98%, Lot no.: IVRTI-NJ) was provided by TCI Europe (Zwikndrecht, Belgium) and used as received.

Zinc diethyldithiocarbamate (ZDEC, Lot no.: A-223K) and high-density polyethylene film (HDPU, Lot no.: C-221) were provided by Hatano Research Institute, Food and Drug Safety Center (Hadano, Japan).

Dulbecco’s Modified Eagle Medium (DMEM/F-12, Lot no.: RNBL4062), PenicillinStreptomycin (Lot No.: 0000205667) and GlutaMAX^TM^ (Lot no.: 2554717) were purchased from ThermoFisher Scientific (Waltham, MA, USA). Tissue Culture Polystyrene (TCPS) 96-well plates were purchased from Becton Dickinson (Le Pont-de-Claix, France).

### 3.2. Nuclear Magnetic Resonance (NMR)

All ^1^H NMR were performed in deuterated chloroform (CDCl_3_) on a Bruker Avance III HD 400 MHz NMR (Billerica, MA, USA) equipped with BroadBand Inverse (BBI) probe. Spectra were processed and visualized with MestReNova software (x64-14.2.1-27684).

### 3.3. Fourier Transform Infrared (FTIR)

The Fourier transform infrared (FTIR) spectra were recorded using attenuated total reflection (ATR) in transmission mode with a ThermoFisher Scientific (Waltham, MA, USA) Nicolet iS50 FT-IR Flex Gold spectrometer equipped with a deuterated triglycine sulfate (DTGS) detector. The characteristic IR absorption bands are reported in cm^−1^.

### 3.4. Thermogravimetric Analyses (TGA)

Thermogravimetric analyses of PEU were performed on a NETZSCH TG 209 F1 Libra^®^ (Selb, Germany) under 50 mL·min^−1^ argon. The protective gas used was argon with a 20 mL·min^−1^ flow. Approximately 10 mg of sample was placed in an alumina crucible and heated from room temperature to 800 °C with a 20 °C·min^−1^ heating ramp.

### 3.5. Differential Scanning Calorimetry (DSC)

Differential scanning calorimetry analyses were carried out using a NETZSCH DSC 3500 Sirius (Selb, Germany). Constant calibration was performed using indium, n-octadecane, n-octane, adamantane, biphenyl, tin, bismuth and zinc standard. Nitrogen was used as the purge gas at 40 mL·min^−1^. Approximately 10 mg of sample was placed in pierced aluminum pans and the thermal properties were recorded between −150 °C and 200 °C at 20 °C·min^−1^ to observe the *T_g_*. Glass transition temperatures were measured on the second heating ramp to erase the thermal history of the polymer. All reported temperatures are averaged values.

### 3.6. Isocyanate Equivalent Weight (IEW) and Hydroxyl Equivalent Weight (HEW)

The IEW and HEW of HDI and PLGA, respectively, were determined by ^1^H NMR titration, using benzophenone as the internal standard. For each compound, three samples were prepared with approximately 50 mg of the sample and 20 mg of benzophenone dissolved in 0.5 mL of CDCl_3_ and analyzed by ^1^H NMR. The IEW and HEW were determined by integration of the signals at 3.30 ppm for HDI, 4.3 ppm and 4.20 ppm for PLGA, and 7.5 ppm for benzophenone (HDI IEW = 79 g·eq^−1^ and PLGA HEW = 1120 g·eq^−1^). An example is given for the HEW calculation of PCL diol, in Equation (1), where N_H eq PCL_ corresponds to the number of hydrogens in alpha to the hydroxyl moiety.

(1) $$\mathrm{HEW}=\frac{m_{\mathrm{PCL}}\times N_{H\;\mathrm{eq}\;\mathrm{PCL}}\times \int_{7.28}^{7.87}\mathrm{benzophenone}}{m_{\mathrm{benzophenone}}\times N_{N\;\mathrm{eq}\;\mathrm{benzophenone}}\times \int_{3.65}^{3.70}\mathrm{PCL}}\;$$

The IEW of the prepolymer was determined with two different methods: ^1^H NMR titration (IEW = 1053 g·eq^−1^) and titration with n-butylamine (ASTM D2572-80 https://www.document-center.com/standards/show/ASTM-D2572, accessed on 15 January 2024) (IEW = 1057 g·eq^−1^). The values were relatively close, and it was decided to determine the IEW by the more convenient ^1^H NMR titration method.

### 3.7. Size Exclusion Chromatography Multi-Angle Light Scattering (SEC-MALS)

SEC-MALS measurements were performed on Agilent 1260 Infinity triple detection SEC set-up (Santa Clara, CA, USA) comprising a Wyatt Optilab MALS detector, and an Agilent differential refractometer. Separation was achieved using 2 PLgel mixed BLS columns (7.5 mm × 300 mm). The eluent was tetrahydrofuran (THF) at 30 °C at a flow rate of 1 mL·min^−1^. The refractive index increment (dn/dc) of PLGA, PCL, the prepolymer and the PEU was obtained as follows: five different concentrations (0.25 mg·mL^−1^, 0.5 mg·mL^−1^, 0.75 mg·mL^−1^, 1 mg·mL^−1^, 1.5 mg·mL^−1^, 2 mg·mL^−1^) of the polymer in THF were injected and the resulting RI signals were plotted as a function of concentration. The dn/dc values of the polymers are given in Table S1 and were used for the MALS analysis of all samples in this work.

### 3.8. Scanning Electron Microscopy (SEM)

A scanning electron microscope (Phenom ProX Desktop, ThermoFisher Scientific, Waltham, MA, USA) was employed to observe the porous structure of the polyurethane scaffolds. Cubic samples (2–3 mm per side) were analyzed. The cross sections of the samples were sputter coated with gold. SEM observation was carried out at 10 kV. The average pore size and analysis of pore size distribution in the samples were determined with ImageJ program. Pore sizes are expressed as mean +/− SD (*n* = 60).

### 3.9. Mechanical Properties

Cubic samples (10 mm per side) were cut for testing. Compressive strengths at 20% deformation and Young’s moduli were determined using Instron^®^ tester model 3366 (Norwood, MA, USA) equipped with 100 N load cells. The measurements were carried out at a crosshead speed of 5 mm·min^−1^. All given values are given as means of three measurements +/− SD. The compression modulus was determined via the stress/strain curve of the material.

### 3.10. Dynamic Mechanical Analyses (DMAs)

Dynamic mechanical analyses (DMAs) were carried out on a Mettler Toledo DMA instrument (Viroflay, France) with STARe software (V 16.30). Compression of samples was performed while heating at a rate of 2 °C⋅min^−1^ from −40 °C to 60 °C, keeping the frequency at 1 Hz.

### 3.11. MALDI-TOF

MALDI-TOF MS were recorded on a Bruker RapiFlex spectrometer operating at the following conditions: nitrogen laser (337 nm), accelerating potential (20 kV) in positive linear ion or reflection mode. In delayed extraction mode, the delay time was ∼300 ns, optimized based on the mass range of the polymer distributions. External calibration was performed using PEG polymer standards. Trans-2-[3-(4-tert-butylphenyl)-2-methyl-2- propenylidene]malononitrile (DCTB) was employed as the matrix. An analyte solution and matrix solution with a concentration of 10 g·L^−1^ in THF (1:4 *v*/*v* analyte-to-matrix solution) were mixed with 1 μL of potassium trifluoroacetate (10 g·L^−1^). An analyte solution (at 40 g·L^−1^ in THF) and matrix solution with a concentration of 10 g·L^−1^ in THF (1:4 *v*/*v* analyte-to-matrix solution) were mixed with 1 μL of potassium trifluoroacetate (10 g·L^−1^) (K^+^ ionization). A total of 1 μL of the resulting mixture was spotted on the MALDI plate for MS analysis. Data were analyzed and normalized using the FlexAnalysis version 3.0 software (Bruker, Billerica, MA, USA).

### 3.12. Viscosity

Viscosity of the PEU solution was measured using an Anton Paar MCR302 instrument (Les Ulis, France), with a cone–plate geometry (50 mm diameter). Measurements were performed at 25 °C at a shear rate between 0.01 and 100 s^−1^. Viscosity value is the mean of three measurements +/− SD.

### 3.13. PLGA Synthesis

PLGA was obtained via the ring-opening polymerization (ROP) of lactide and glycolide with stannous octoate Sn(Oct)_2_ initiated with 1,3-propanediol (Figure S1). D,L-lactide and glycolide were dried under vacuum overnight prior to use. Under nitrogen, 22.15 g of D,L-lactide (0.15 mol) and 17.85 g of glycolide (0.15 mol) were charged into a rigorously dried 100 mL nitrogen flask immersed into a preheated oil bath (T = 130 °C). After observing the clear melting of monomers, 1.52 g of 1,3-propanediol (0.02 mol) and 0.08 g of the catalyst Sn(Oct)_2_ (0.5% wt, 2.10^−4^ mol) were introduced. The reaction was allowed to proceed for 3 h at 130 °C. After cooling to room temperature, the products were dissolved in 50 mL of THF and then precipitated in 500 mL of cold heptane. The polymer was collected by filtration and dried at 25 °C under vacuum for 24 h.

A 50:50 ratio of lactic:glycolic units was calculated by ^1^H NMR according to Equation (2), where I_5.2 ppm_ and I_4.8 ppm_ correspond to the integration values of the signals of lactic acid and glycolic acid, respectively.

(2) % of lactic acid=1001+I5.2 ppmI4.8 ppm2 

^1^H-NMR (400 MHz, CDCl_3_, δ): 5.2 (m, 16H, H_a_), 4.8 (m, 34H, H_b_), 4.4 (m, 1H, H_e_), 4.3 (s, 2H, H_f_), 4.2 (m, 4H, H_g_), 2 (m, 2H, H_c_), 1.6 (m, 56H, H_d_) (Figure S1).

### 3.14. PCL di-NCO Prepolymer Synthesis

A total of 17 g of HDI (IEW = 79 g⋅eq^−1^, 4 eq) was introduced into a rigorously dried 100 mL, two-neck, round-bottom flask. The system was purged with nitrogen for 10 min and then heated up to 80 °C. A total of 50 g of PCL (HEW = 1135 g·eq^−1^) was solubilized in 60 mL of toluene and added dropwise with a syringe driver for one hour (60 mL·h^−1^) with magnetic stirring. The mixture was stirred for an additional 3 h. Then, the prepolymer (IEW = 1053 g·eq^−1^) was precipitated in 800 mL of cold pentane. The prepolymer was collected by filtration and dried at 40 °C under vacuum for 48 h. It was stored at −20 °C.

^1^H-NMR (400 MHz, CDCl_3_, δ): 4.1 (m, 4H, H_i_), 4.0 (m, 37H, H_h_), 3.2 (t, 4H, H_k’_), 3.1 (m, 4H, H_m_), 2.2 (t, 37H, H_n_), 1.6 (m, 96H, H_p_), 1.5 (m, 7H, H_s,_ H_j_), 1.3 (m, 48H, H_r_, H_l_) (Figure S2).

### 3.15. Poly(ester-urethane) Synthesis

The extension of the PCL di-NCO prepolymer with PLGA was performed in a reactor. A total of 7 g of PLGA (HEW = 1120 g⋅eq^−1^) was added into a dry glass reactor purged with nitrogen, with 10 mL of distilled dioxane. The reactor was heated to 100 °C, and 8 g of PCL di-NCO prepolymer (IEW = 1053 g eq^−1^) was added, with tin(II) 2-ethylhexanoate (0.5% wt). The system was reacted with a mechanical stirring blade at this temperature for 1 h, until disappearance of the isocyanate band at 2300 cm^−1^. Following the polyaddition, distilled dioxane was slowly added to control the viscosity of the mixture and allow for the transfer and use of the PEU. The polymer was purified by precipitation in 80:20 diethylether:EtOH, with a final 84% yield.

### 3.16. Scaffold Preparation

Porous polymer scaffolds were prepared by SC/PL with NaCl salt as porogen agent. Different ratios of salt crystals/PEU were tested (1, 3, 5 and 7 g per g of PEU). In a typical experiment, 3 g of PEU was dissolved in 8 mL of dioxane. A total of 15 g of NaCl crystals sieved to 100–300 µm were then added to the polymer solution. This mixture was vigorously mixed and poured into a mold. The filled mold was immersed into liquid nitrogen, after which the mixture was left to evaporate in a desiccator at room temperature under vacuum with a controlled flowrate for one night. The foam was washed in distilled water at room temperature until clearance of salt crystals.

To characterize the foam obtained, the porosity was calculated according to Equation (3), where m is the mass of the scaffold (g), V the volume of the scaffold (cm^3^) and ρ_PEU_ is the volumic mass of the PEU (g/cm^3^). Porosity values are the mean of three measurements +/− SD.

(3) $$P=1-\frac{m}{\rho_{\mathrm{PEU}}V}\;\times 100\;$$

### 3.17. Cytotoxicity Study

The cytotoxicity of the PEU was investigated through the quantification of adenosine triphosphate (ATP). The results were characterized with Spectramax i3x (Molecular Devices). Cells and control polymers were chosen in accordance with ISO-10993 guidelines.

L929 murine fibroblasts: The cytotoxicity of PEU film was investigated on murine fibroblast cell line, L929 (NCTC-Clone 929, ECACC 85011425). L929 cells were cultured at 37 °C under humidified 5% CO_2_ in Dulbecco’s Modified Eagle Medium 4.5 g·L^−1^ D-glucose supplemented with 2 mM L-glutamine, 5% *v*/*v* Fetal Bovine Serum and 100 U per mL of penicillin and 100 μg·mL^−1^ of streptomycin.

CellTiter-Glo^®^ Luminescent Cell Viability assay (reference G7571, Lot No.: 0000497159): PU film containing 0.1% ZDEC, batch A-202K, was used as positive reference material (RM-A) and HDPE, batch C-221, was used as negative RM (RM-C). All control materials were supplied by Hatano Research Institute, Food and Drug Safety Center 729-5 Ochiai, Hadano, Kanagawa, Japan. PEU film, both positive and negative RMs, were irradiated at λ = 254 nm for 2 min, twice on each face for decontamination. Specimens were immersed in the culture medium (1 mg·mL^−1^) and incubated for 72 h at 37 °C. The cells were seeded into a 24-well plate at a density of 60,000 cells per well and were incubated overnight at 37 °C under humidified 5% CO_2_. Then, the medium in contact with the materials was extracted and 100 µL was incubated with cells for an additional 24 h. The number of viable cells was obtained by a CellTiter-Glo^®^ assay, based on quantification of the present ATP, which represents metabolically active cells. CellTiter-Glo^®^ reagent was added to each well and the plate was placed at room temperature for 10 min to stabilize the luminescent signal before reading. The background, corresponding to wells free of cells, was subtracted from each triplicate mean.

Statistical analysis: The data are shown as the means ± standard deviation (SD), and the statistical difference of the results was evaluated using R software (V 3.6.1). Non-parametric data results were analysed using Kruskal–Wallis test followed by Dunn post hoc test (threshold of significance * *p* < 0.05).

### 3.18. In Vitro Degradation

The kinetics of degradation were studied in vitro in accelerated conditions using HCl aqueous solution (0.1 M, pH = 1) at constant temperature (37 °C) and under continuous stirring (100 rpm). Scaffold samples were cut into cubic shape, weighed (w_dry,t0_) and incubated in 0.75 mL of HCl solution (pH = 1). The samples were removed from the medium at specific time points, washed with distilled water, carefully wiped and then dried to constant weight (w_dry,t_). Degradation was monitored by determination of the weight loss and molecular weight decrease in the PEU. The remaining weight was calculated from Equation (4) and the remaining molecular weight from Equation (5).

(4) $$\mathrm{remaining}\;\mathrm{mass}\;\left(t\right)\left(\%\right)=\left(1-\frac{w_{\mathrm{dry},t0}-w_{\mathrm{dry},t}}{w_{\mathrm{dry},t0}}\right)\times 100\;$$

(5) $$\mathrm{remaining}\;\mathrm{Mn}\left(t\right)\;\left(\%\right)=\left(1-\frac{{\mathrm{Mn}}_{0}-{\mathrm{Mn}}_{t}}{{\mathrm{Mn}}_{0}}\right)\times 100\;$$

## 4. Conclusions

The objectives of this work were to design porous degradable scaffolds with mechanical and degradation properties suitable with meniscal repair applications. To this end, we designed and prepared a high-molecular-weight (*M_n_*~87 kg·mol^−1^) poly(ester-urethane) (PEU) in two steps using diisocyanate prepolymer PCL di-NCO and PLGA as a chain extender. By optimizing the critical parameters (PEU and salt concentration, solvent evaporation technique), the PEU has been efficiently processed into a 3D porous scaffold by applying a SC/PL process. Increasing the salt concentration led to a Young’s modulus decrease (from 35 MPa to 6 MPa) and porosity increase (from 36 to 72%) to reach values close to the commercial meniscal implant Actifit^®^. The SEM confirmed that the scaffolds exhibited relatively uniform interconnected pores with sizes around 150 µm. An accelerated degradation study proved that incorporating PLGA into the scaffolds could greatly accelerate the degradation compared to the all-PCL-based commercial gold standard. Finally, a cytotoxicity study confirmed the biocompatibility of the PEU, with a 90% cell viability. Overall, this study confirms that the PEU scaffolds hold promise for meniscus repair, which should be further confirmed by in vivo preclinical studies to evaluate their performance in terms of tissue integration and regeneration.

## Figures and Tables

**Table 1:** Summarized data of the four foams synthetized with a different salt concentration and Actifit^®^. Sample size for experiments performed on Instron was 10 mm^3^ (*n* = 3). Sample size for DMA experiments was 2 mm^3^ (*n* = 3).

Salt Quantity (g/1 g PEU)	1	3	5	7	Actifit^®^
Pores size (µm)	111 ± 91	164 ± 40	151 ± 51	155 ± 48	240 ± 30
Porosity (%)	36 ± 0	52 ±1	57 ± 2	72 ± 2	53 ± 4
Ey (Mpa) at 25 °C	35 ± 1	33 ± 6	14 ± 1	12 ± 1	6 ± 1
E′ (MPa) at 25 °C	38 ± 5	25 ± 3	21 ± 0	8 ± 1	2
E′ (MPa) at 37 °C	4.5 ± 0.8	4.1 ± 2.3	2.8 ± 0.2	1.5 ± 1.1	1.3
E″ (MPa) at 37 °C	2.5 ± 0.8	2.7 ± 1.9	1.4 ± 0.1	1.0 ± 0.1	0.1
T_α_ (°C)	24	28	24	25	−43
*T_g_* (°C)	45	47	49	49	−50

## Data Availability

The raw data are available on request from the corresponding author.

## Supplementary Materials

The following supporting information can be downloaded at https://www.mdpi.com/article/10.3390/molecules29040766/s1, Figure S1: Idealized PLGA structure; Figure S2: Idealized prepolymer PCL di-NCO structure; Figure S3: MALDI-TOF of PLGA 50:50 with (a) the scale from 600 to 300 *m*/*z* and (b) a zoom in from 1400 to 1500 *m*/*z*; Figure S4: ATG of the polymers; Figure S5: Chromatogram of Actifit^®^ (SEC MALS-THF); Figure S6: Viscosity curve of the PEU in solution in dioxane; Figure S7: DSC thermograms of the foams prepared with different amount of porogen agent (g salt/g polymer); Figure S8: Pore size repartition of foam with 5 g of salt/g PEU and Actifit^®^; Table S1: dn/dc values obtained for the polymers.
