# Supplementary material for: Evaluation of Porous (Poly(lactide-co-glycolide)-co-(ε-caprolactone)) Polyurethane for Use in Orthopedic Scaffolds
Source: Molecules. 2024 Feb 7;29(4):766. doi: 10.3390/molecules29040766 (PMC10891616; doi:10.3390/molecules29040766)
Supplement: Supplementary file 1 [file molecules-29-00766-s001.zip › molecules-2849871-supplementary.pdf]

Supplementary material

Evaluation of porous (poly(lactide-*co*-glycolide)-*co*-(ε-caprolactone)) polyurethane for use in orthopedic scaffolds

Gaëlle Savin [1,2,3], Océane Sastourne-Array [2], Sylvain Caillol [1*], Audrey Bethry [2], Michel Assor [3], Ghislain David [1], Benjamin Nottelet [2,4*]

1 ICGM, Univ Montpellier, CNRS, ENSCM, Montpellier, France gaelle.savin@enscm.fr (G.S.); oceane.sastourne-arrey@etu.umontpellier.fr (O.S-A.); ghislain.davivd@enscm.fr (G.D.)
2 Polymers for Health and Biomaterials, IBMM, Univ Montpellier, CNRS, ENSCM, Montpellier, France
3 Arthrocart Biotech, Marseille, France
4 Department of Pharmacy, Nîmes University Hospital, Univ Montpellier, 30900, Nimes, France

* Correspondence: sylvain.caillol@enscm.fr (S.C.); benjamin.nottelet@umontpellier.fr (BJ)

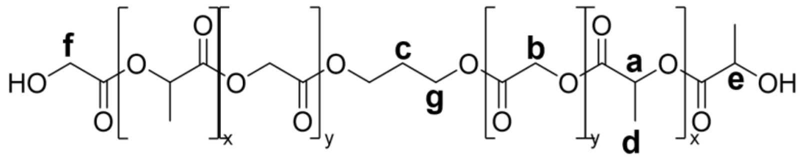

Figure S1. Idealized PLGA structure.

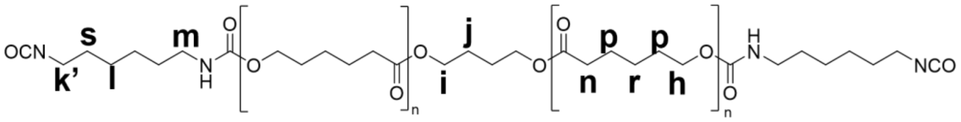

Figure S2. Idealized prepolymer PCL di-NCO structure.

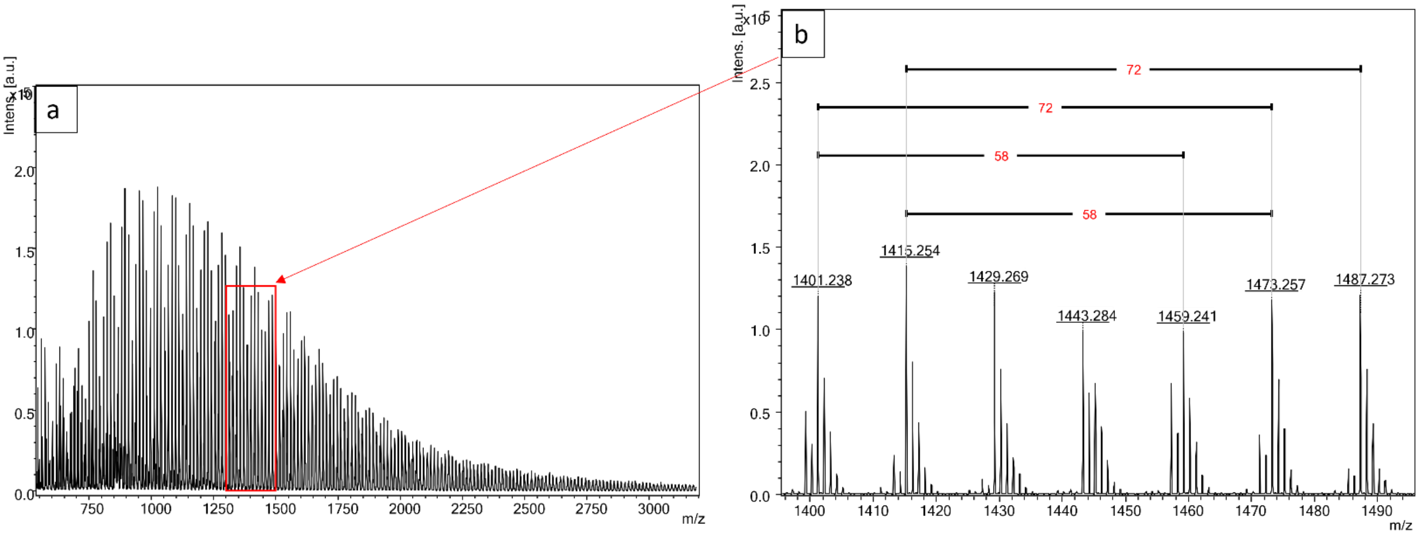

Figure S3. MALDI-TOF of PLGA 50:50 with a) the scale from 600 to 300 m/z and b) a zoom in from 1400 to 1500 m/z.

Table S1. dn/dc values obtained for the polymers

	PLGA 50	Prepolymer	PEU
dn/dc (ml/g)	0.044 ± 0.003	0.079 ± 0.003	0.069 ± 0.004
R^2	0.9894	0.9957	0.9957

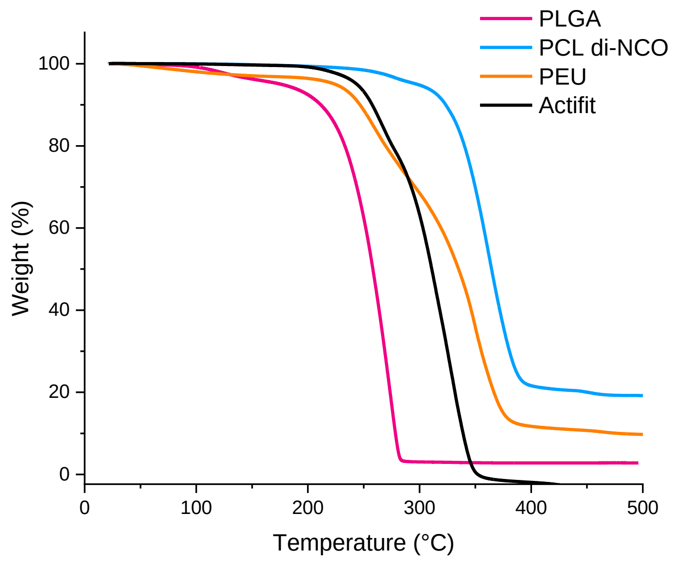

Figure S4. Thermogravimetric analysis of the polymers.

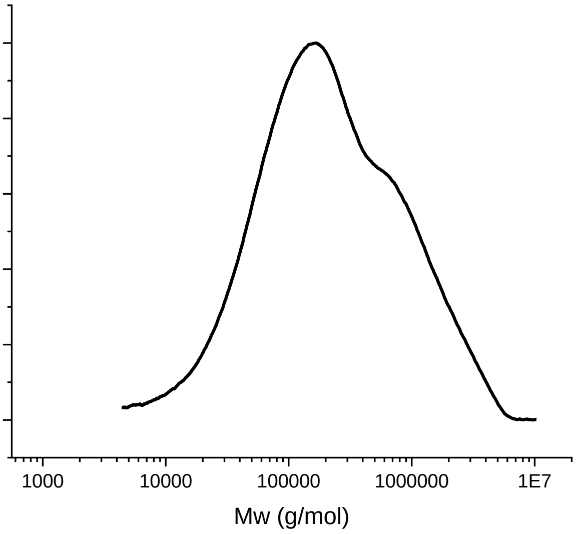

Figure S5. Chromatogram of Actifit® (SEC MALS-THF).

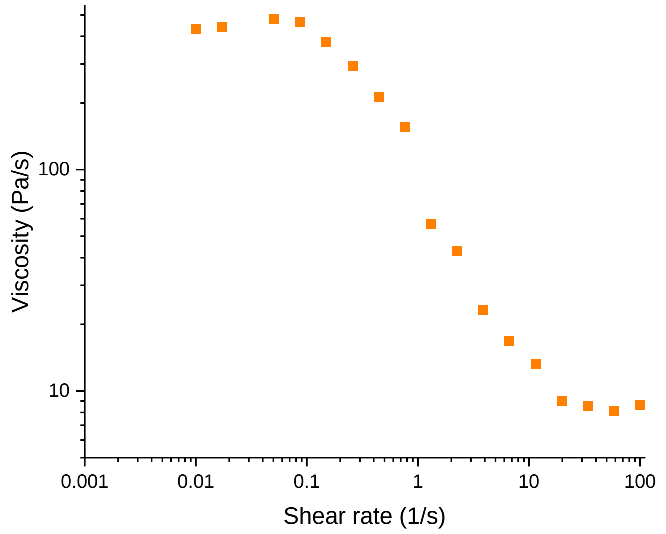

Figure S6. Viscosity curve of the PEU in solution in dioxane.

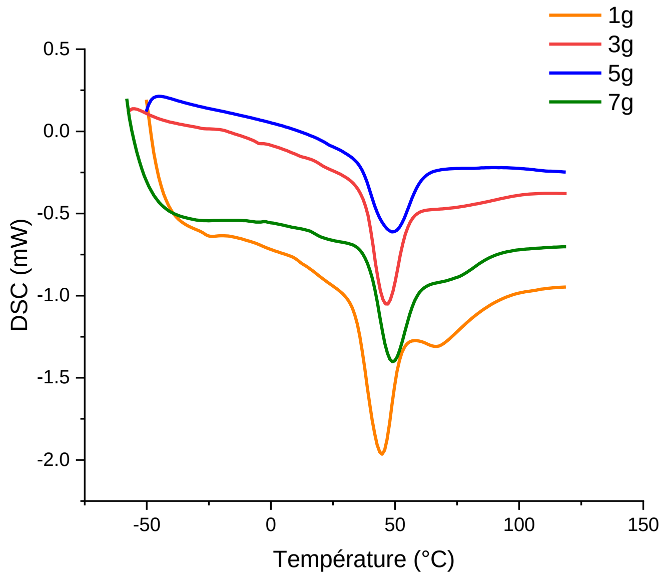

Figure S7. Differential scanning calorimetry thermograms of the foams prepared with different amount of porogen agent (g salt/g polymer).

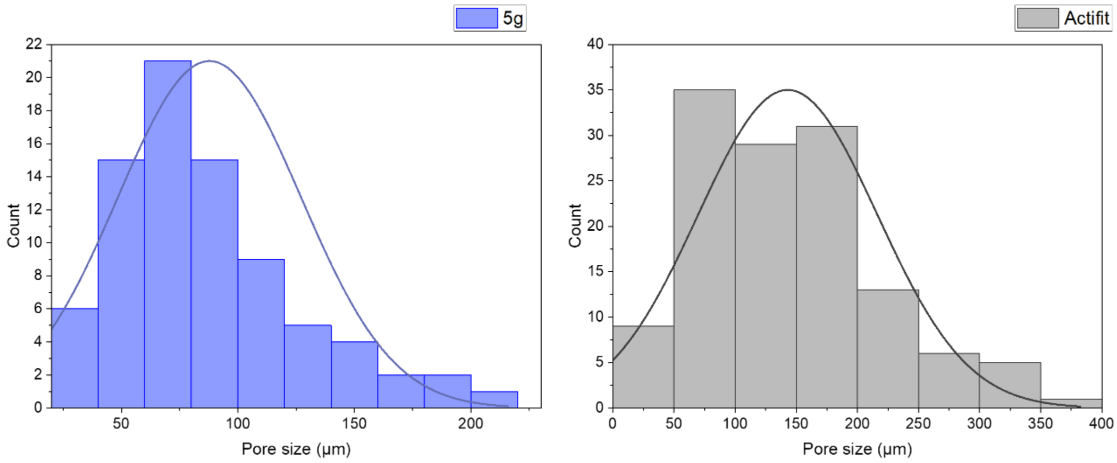

Figure S8. Pore size repartition of foam with 5g of salt/g PEU and Actifit®.
